# Detection and differentiation of antinuclear antibodies in serum of dengue suspected patients with or without systemic autoimmune disease in Kolkata, India

**DOI:** 10.1080/21505594.2024.2400553

**Published:** 2024-09-16

**Authors:** Rajendra Prasad Chatterjee, Shilpa Chatterjee, Debsopan Roy, Shyamalendu Chatterjee, Nilanjan Chakraborty

**Affiliations:** a Virus Research Laboratory, ICMR-National Institute of Cholera and Enteric Disease, Kolkata, India; b Department of Pharmaceutical Technology, School of Health and Medical Sciences, Adamas University, Barasat, Kolkata, India

**Keywords:** Dengue virus, antinuclear antibodies, systemic autoimmune disease, non-rheumatic disease, immunofluorescence assay, line immunoassay

## Abstract

The pathophysiology of dengue may be influenced by antibodies released during infection. Several autoimmune diseases are accompanied by antinuclear antibodies (ANAs) but 8–10% of the general population have positive ANA tests. To test the hypothesis that an ANA-positive test indicates an immune dysregulated state that modifies the risk for certain clinical disorders in people with or without an autoimmune disease, we examined the various ANA profiles and their relationships to various autoimmune disorders, as well as the severity of these relationships, in patients infected with dengue fever. Enzyme-linked immunosorbent assay (ELISA) and reverse transcription-polymerase chain reaction (RT-PCR) methods were used. Indirect immunofluorescence assay (IIFA) and line immunoassay (LIA) were performed to detect and differentiate the ANAs among dengue infected patients. Out of 135 dengue virus-positive patients, 94.07% were positive by ELISA and 5.93% positive by RT-PCR method. ANAs by IIFA and LIA were detected in 54.8% and 18.5% of the dengue positive patients, respectively, and 10.3% and 7.1% of the 126 dengue negative patients, respectively. This study showed that dengue was associated with an increased risk of autoimmune myositis and mixed connective tissue disease (MCTD), a rare complication of dengue. The risk of other autoimmune diseases did not seem to increase after DENV infection.

## Introduction

Recent decades have seen a sharp rise in the worldwide incidence of dengue, an alarming virus spread by mosquitoes [1]. Dengue virus (DENV) infection poses a significant public health threat and has been reported in Southeast Asia, Africa, the Americas, Europe, the Western Pacific, and the Eastern Mediterranean. It is estimated that four billion individuals are at risk of contracting DENV, with tropical and subtropical regions bearing the greatest burden [2]. Estimates suggest that 390 million people contract DENV annually, with 96 million experiencing varying degrees of symptoms [3]. DENV is determined to be endemic in more than 100 countries. In many parts of the world, especially Southeast Asia and the Indian subcontinent, DENV is one of the main causes of considerable morbidity and economic burden. Prior research has shown that antibodies generated during DENV infection can reacts with several human self-antigens, including platelet cells, integrin, and plasminogen [4,5]. These autoantibodies may lead to autoimmunity and may also have a role in the clinical development of severe dengue [4,6]. While previous beliefs held that autoimmunity resulting from dengue infections would only last for a brief period, a recent study in Cuba discovered that over 50% of patients continued to experience chronic symptoms two years following their initial infection. Additionally, these patients exhibited fluctuating antinuclear antibody levels and other autoimmune markers [7]. Additionally, according to certain case reports, dengue may be linked to several autoimmune illnesses [8–12]. While symptomatic infections caused by DENV infections typically follow a straightforward course, there has been a recent increase in the identification of uncommon symptoms and complications. The presence of antinuclear antibody (ANA) is a primary indicator of autoimmune disease and is utilized for illness diagnosis, prognostic assessment, and activity monitoring. ANA-associated rheumatic disorders (AARDs) are a group of diseases that are specifically associated with various autoantibodies. These diseases include systemic lupus erythematosus (SLE), Sjögren’s syndrome (SjS), systemic sclerosis (SSc), and mixed connective tissue disease (MCTD) [11,12]. To diagnose AARDs, it is crucial to investigate the ANA profile, which includes specific antibodies against various targets like Smith (Sm) antigen and double-stranded DNA (dsDNA) for SLE, Ro60 and SS-B/La for SjS, Scl-70 and RNA polymerase III for SSc, and U1-ribonucleoprotein (RNP) for MCTD [13]. ANA immunofluorescence patterns are commonly utilized to assess autoantibody specificities. The ANA profile of extractable nuclear antigen (ENA) specificities is usually determined through line immunoassay (LIA), while the ANA test is conducted via IIFA with HEp-2 cells. Thanks to its high sensitivity, ANA IIFA is frequently employed as a screening test for ANA. This is because it can analyze antibody titers and immunofluorescence patterns. However, additional testing is often necessary to confirm antibody specificity. LIA is extensively used to evaluate antibody specificity and can quantitatively measure antibody titers with high specificity. Moreover, LIA is beneficial for simultaneously screening and typing different antibodies, as it can measure many antibodies in less time. Another study found evidence that dengue affects the clinical disease after the acute phase of sickness, including a case of lupus nephritis that developed in the later phases of the infection [10]. In cases of dengue infection, the pathogenesis of lupus nephritis is significantly impacted by host factors. This complex process results from a combination of the virus’s pathogenic effects and the host’s immune system’s response to the virus. Notably, in 2012, Rajadhyaksha and Mehra from India were the first to document a case of dengue fever progressing to lupus nephritis in international literature [14]. Our team has discovered another case of MCTD accompanied by autoimmune myositis, which occurred after a patient had contracted dengue fever. Extensive research has been conducted globally, shedding light on various factors related to the human pathophysiology of dengue. Despite the wealth of information on the subject, there are still areas where knowledge gaps exist, hindering a complete understanding of the disease’s etiology and severe manifestations. In this article, the latest discoveries related to the pathogenesis of dengue virus infection are presented. A comprehensive summary of all the factors that contribute to the severe clinical manifestations of the disease is also provided. Additionally, this piece offers a brief overview of current advancements in dengue pathogenesis research as well as the role of various biomarkers in serving as early indicators of disease severity. Dengue viral infections are known for their acute and sometimes severe manifestations. Recent studies suggest that dengue can trigger the production of autoantibodies, which may contribute to the development of autoimmune diseases [4,5]. Considering various factors, it is possible that the pathophysiology of dengue could be impacted by autoantibodies. However, a thorough screening of autoantibody reactivities during the acute phase of DENV infection has yet to be conducted. To address this, we utilized a line immunoassay to test the serum of DENV infected patients with varying degrees of illness severity. This method can detect many autoantibodies on a nitro cellular membrane. Our study takes an exploratory approach in examining the autoantibody profiles in DENV infection and their potential correlation with disease severity.

## Materials and methods

### Study area

This retrospective study was conducted in Virus Research Laboratory, Indian Council of Medical Research (ICMR)-National Institute of Cholera and Enteric Diseases, Kolkata, India, during February 2021 - February 2024. This study was authorized by Ethics Committee of ICMR- NICED, Kolkata (Institutional memo no: ICMR/VU/57-DBT). Well-informed permission was acquired from all discrete contributors included in this study. Written consent to publish has been received from the participants.

### Sample collection and storage

Sample collection and storage was done as per laboratory protocol [15]. Details are mentioned here briefly. Specimens were collected randomly from 261 dengue suspected patients, and the study was planned to be non-discriminatory in terms of age or gender. Patients who visited the fever unit with illnesses other than febrile illnesses and patients who declined to participate in the study were both excluded from it. Approximately, 2-3 ml of blood samples was collected by the laboratory personnel from the patients having fever along with other possible history of illness from the fever clinic unit of different medical colleges and hospitals. Dengue cases were classified as dengue fever according to 1997 guidelines of World Health Organization (WHO) [16]. Infection with DENV was confirmed if one of the following tests was positive, (1) DENV specific real-time RT-PCR, (2) DENV IgM detection by PanBio Dengue IgM Capture ELISA.

Along with the blood samples, clinical and demographical data of patients were collected by the health-care workers at the time of patients’ visit and sent to our laboratory for further analysis. Maintaining cold chain, all the samples were transported to our virology laboratory for the confirmation of the infection, if any. The centrifugation was used to isolate serum from the specimens at 3000 g for 10 min at 4 °C. Serums were stored for serological and molecular tests at −20°C and −80°C, respectively, in aliquots, until further used.

### Serological detection of DENV

On purpose of serological detection, all the DENV suspected samples were subjected to ELISA to detect Anti-DENV-IgM antibody. Kit inserted protocol was used from commercially available kits having 94.7% sensitivity and 100% specificity from PanBio Dengue IgM Capture ELISA (PanBio Kit, Alere, Waltham, MA, USA) [17]. We also received DENV IgM ELISA kits from ICMR-National Institute of Virology to use as reference having 95% sensitivity and 98% specificity. Optical Density was measured at 450 nm using Thermo scientific multi scan EX reader.

## Molecular detection of DENV

### RNA extraction and RT-PCR

All the DENV suspected samples were subjected to RNA isolation using a QIAamp viral RNA isolation kit (Qiagen, GmbH, Hilden, Germany) as per kit insert protocol. After RNA extraction, a one-step RT-PCR targeting the DENV non-structural protein 2A (NS2A) gene was performed using commercially available RT-PCR kit from Chromous Biotech (Chromous Biotech Pvt. Ltd., Bangalore, India). The primer sequences used in the template formation were DENV-FP (5″-CACAACCCATGGAACACAAATACTCG-3′) and DENV-RP (5′-CATATCGGCATGAACTGCCTTGCTGTC −3″). The assay was carried out in a Bio-Rad thermal cycler, starting with the cDNA synthesis (42°C for 15 min), followed by inactivation (94°C for 30 sec), and finally 35 PCR cycles, which include denaturation at 94°C for 30 sec, annealing at 50°C for 30 sec, and expansion at 72°C for 30 sec. The final elongation stage was fixed out for 7 minutes at 72°C. The final PCR products were run in a gel electrophoresis system (made by Bio-Rad) for 45–60 minutes at 80–100 volts in a 2% agarose gel before being exposed to UV light in a gel imager. For each positive sample, the amplified products were seen as bands that were approximately 280 base pair (bp) in size.

### Detection of antinuclear antibodies

#### IIFA (screening test)

We used the Immunoconcepts HEp-2000® ANA Test System with transfected mitotic* human epithelioid cells (HEp-2), represents an advanced immunofluorescent system for detection of ANA [18,19]. It is an indirect fluorescent antibody test system (HEp-2000® fluorescent IgG ANA-Ro IVD kit by Immunoconcepts, USA) for the semi-quantitative detection of ANA IgG in serum among all the dengue suspected patient by manual fluorescent microscopy (Ayrus Epi microscope with LED epifluorescence technology by Medsource Ozone Biomedicals Pvt. Ltd., India). Samples were incubated with antigen substrate to allow specific binding of autoantibodies to cell nuclei. If ANAs are present, a stable antigen-antibody complex is formed. After washing to remove non-specifically bound antibodies, the substrate is incubated with an anti-human antibody conjugated to fluorescein. When results are positive, there is the formation of a stable three-part complex consisting of fluorescent antibody bound to human ANA, which is bound to nuclear antigen. This complex can be visualized with the aid of a fluorescent microscope. In positive samples, the cell nuclei will show an apple-green fluorescence with a staining pattern characteristic of the nuclear antigen distribution within the cells. If the sample is negative for ANA, the nucleus will not show a clearly discernible pattern of nuclear fluorescence. 200X total magnification is used for screening positive/negative and titer end-point determination, while 400X total magnification is used for pattern recognition and viewing mitotic cells. Fluorescent intensity (1+, 2+, 3+ and 4+) has been semi-quantitated against each IIFA positive samples by following the guidelines for fluorescent antibody reagents established by the Centres for Disease Control and Prevention, Atlanta, Georgia (CDC).

#### LIA (confirmatory test)

We have performed a line immunoassays test (IMTEC-ANA-LIA-XL IVD kit by Human, Germany) for the confirmation and differentiation of the specific autoantibodies among the positive ANA IIFA samples. The test is based on the principle of the line immunoassays that are produced by coating nuclear and associated cytosolic antigens as lines on a nitrocellulose membrane. Bound autoantibodies are made visible by using HRP-conjugated secondary antibodies and a LIA-specific substrate. Test was performed using manufacturer’s protocol among all the IIFA positive samples for the qualitative determination of 18 IgG class antibodies in one assay against dsDNA, Nucleosome, Histone, SmD1, PCNA, PO (RPP), SS-A/Ro60, SS-A/Ro52, SS-B/La, CENP-B, Scl70, U1-snRNP, AMA M2, Jo-1, PM-Scl, Mi-2, Ku and DFS-70 [20]. These autoantibodies are well-documented markers for autoimmune diseases. All the Anti-nuclear antibodies associated with different autoimmune diseases are mentioned in Supplementary Table S1. For the validation and interpretation of the LIA strips Huma Scan FA software (Ref: ITC81000) has been used and indexed values were noted with the help of Canon Cano-Scan LiDE 220 scanner against each ANA LIA positive samples.

## Statistical analysis

SPSS version 25.0 (IBM Corp. Released in 2017. IBM SPSS Statistics for Windows, Version 25.0. Armonk, NY: IBM Corp.) was utilized for statistical analysis. To test the continuous variables between the means of study groups, either Student’s t-test or Welch’s test was used. For categorical variables, Chi-square and Fischer’s exact tests on the groups were conducted. To perform multivariate analysis, multiple logistic regression model. Effects were deemed significant if *p* < 0.05.

## Results

**Figure 1.**
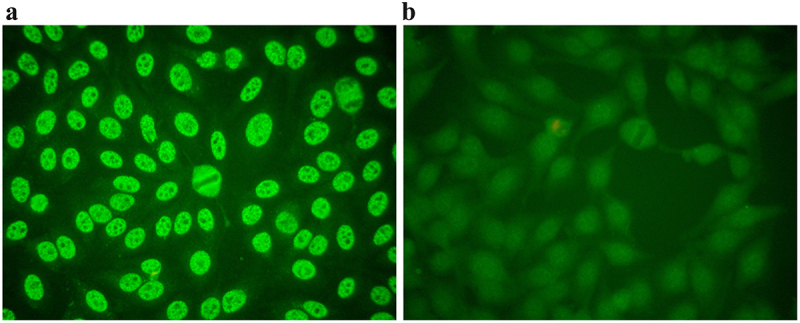
Detection of ANA using IIFA under fluorescence microscope in dengue positive patient. (a) ANA positive by IIFA. (b) ANA negative by IIFA.

**Figure 2.**
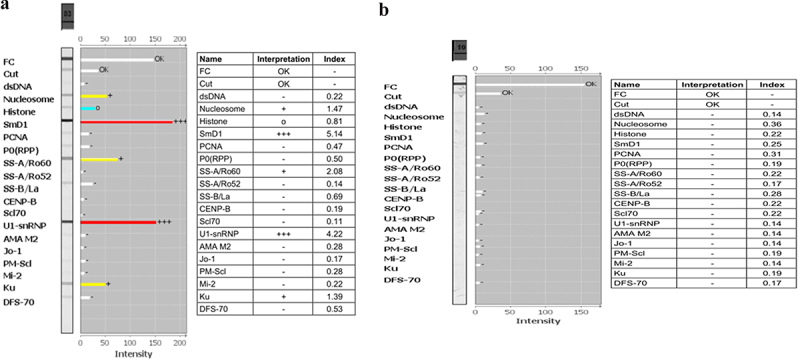
Diagnosis of antinuclear antibody in serum of dengue positive patients using line immunoassay (LIA). (a) ANA profile positive by LIA. (b) ANA profile negative by LIA.

A total of 261 patients were included in this study, including 135 laboratory-confirmed dengue patients and 126 suspected dengue negative patients. Among 135 DENV positive patients, 127 was ELISA positive and 8 showed positivity by RT-PCR method. All 261 DENV suspected samples were tested for ANA IIFA and LIA. Out of 135 DENV positive samples, ANA IIFA positive and negative were 74 and 61, respectively (Figure 1a,b) and ANA LIA positive and negative were 25 and 110, respectively (Figure 2a,b). Out of 126 DENV negative samples, ANA IIFA positive and negative were 13 and 113, respectively, and ANA LIA positive and negative were 9 and 117, respectively. The mean follow-up period was 6–7 months for both the dengue positive and negative patients.

**Figure 3.**
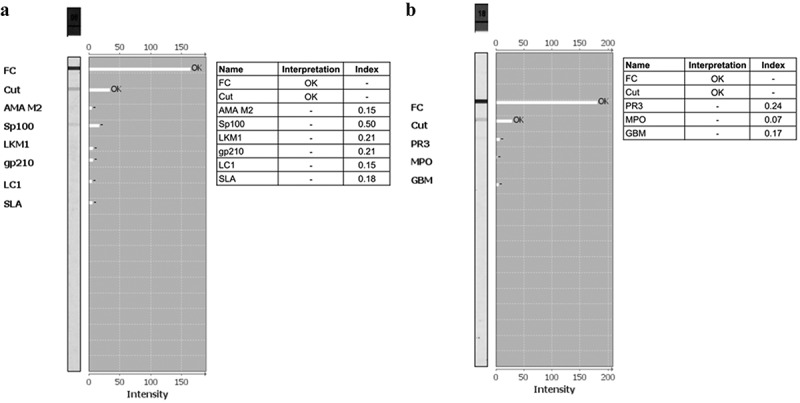
Diagnosis of autoimmune hepatitis and autoimmune vasculitis among dengue positive patients using LIA. (a) ANA liver profile negative by LIA. (b) ANA vasculitis profile negative by LIA.

**Figure 4.**
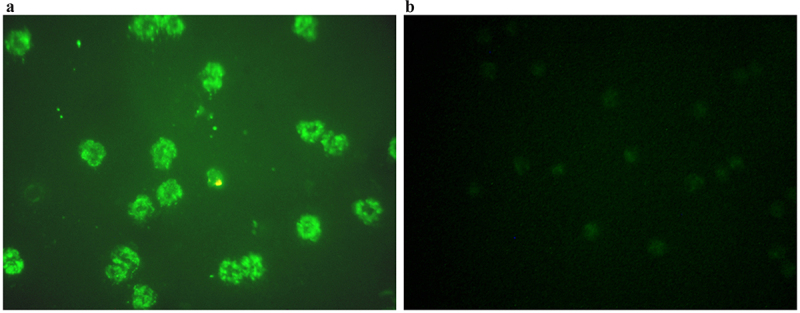
Detection of ANCA using IIFA under fluorescence microscope in dengue positive patient. (a) ANCA positive by IIFA. (b) ANCA negative by IIFA.

We observed that all the ANA LIA positive patients in both DENV positive and negative groups were positive to ANA IIFA. Additionally, all the ANA IIFA positive patients in the current study tested negative for autoantibodies linked to autoimmune hepatitis and vasculitis in LIA (Figure 3a,b), but only one ANA IIFA positive patients showed positive to antineutrophilic cytoplasmic antibody (ANCA) by IIFA, which is responsible for autoimmune vasculitis (Figure 4a,b). It will be worthy to mention that one individual was positive to d-gliadin and tTG autoantibodies, which caused autoimmune gastritis (Supplementary Figure S1). Presence of only DFS70 antibody is also found in one patient, and used as a useful biological marker for identifying positive ANA carriers who do not develop SAD (Supplementary Figure S2). These findings suggest a strong association between dengue infection and the production of autoantibodies commonly linked to autoimmune diseases.

The baseline characteristics for dengue and non-dengue cohorts showed no meaningful difference except that a higher percentage of dengue patients suffered with myalgia, arthralgia, body ache and skin rashes (Table 1). The distribution of comorbidities was not significantly different between the two groups. Univariate Comparison of categorical parameters between dengue positive and dengue negative patients showed that there is no significant correlation of the dengue seropositivity status between the dengue positive and negative patients in respect to the age group (*p* = 0.065). However, in case of clinical symptomatic strata, it has been observed that myalgia (*p* = 0.003) and arthralgia (*p* = 0.011) are the two significant clinical parameters that have been observed to be positively coordinated with the dengue-positive status. Both dengue positive and negative patients were classified into three separate groups according to the range of fever that is Mild fever: <100, Medium fever: 101–103, High fever: >103. In all observation, significant differences among the fever status between dengue positive and negative groups were not observed. Other than the clinical parameters from some physical manifestations like body ache (*p* = 0.005) and skin rashes (*p* = <0.001) were found to be significantly much more prominent in dengue positive patients. For both initial screening test ANA IIFA and confirmatory test ANA LIA, it has been observed that dengue positive patients were significantly showed much more positive coordination with the test results (ANA IIFA *p* < 0.001 and ANA LIA *p* = 0.009) (Table 1).

**Table 1.** Univariate comparison of demographic and laboratory characteristics between the patient groups with or without positive DENV infectivity.

Characteristics	Total sample (n = 261) (%)	DENV Positive (n = 135) (%)	DENV Negative (n = 126) (%)	P value
**Sex of Study population**				0.611
Male	162 (62.1%)	86 (63.7)	76 (60.3)	
Female	99 (37.9%)	49(36.3)	50(39.7)	
Age group				0.065
Group-I: <20	3(1.14)	1(33.3)	2(66.6)	
Group II: 21-40	115(44)	65(48.1)	50(39.6)	
Group III: 41-60	116(44.4)	51(37.7)	65(51.5)	
Group IV: 60-80	27(10.3)	18(13.3)	9(7.1)	
**Clinical symptoms**				
Headache	194(74.3)	110(81.4)	84(66.6)	0.230
Nausea	214(81.9)	108 (80)	106(84.1)	0.423
Myalgia	194(74.3)	111(82.2)	24(17.8)	0.003
Arthralgia	178(68.19)	102(57.3)	76(42.7)	0.011
Sore throat	47(18)	26 (19.25)	21(16.6)	0.631
Vomiting	7(2.6)	3(2.2)	4(3.17)	0.635
Fever				
Mild fever: <100	56(21.45)	11(8.14)	45(35.7)	
Moderate fever: 101-103	135(51.7)	71(52.5)	64(50.7)	
High fever: >103	70(26.8)	53(39.2)	17(13.4)	
Body ache	179(68.58)	103(57.5)	76(42.6)	0.005
Abdominal pain	42(16.09)	20(14.81)	22(17.4)	0.615
Retro orbital pain	37(14.9)	16(11.8)	21(16.6)	0.290
Skin rash	112(42.9)	85(67.4)	27(20)	<0.001
**Testing done**				
ANA IIFA	87(33.3)	74(54.8)	13(10.3)	<0.001
ANA LIA	34(13.2)	25(18.5)	9(7.1)	0.009

Abbreviations: DENV, dengue virus; ANA, autoantibodies for nuclear antigens; IIFA, Indirect immunofluorescence assay; LIA, line immune assay.

Comparisons of ANA IIFA tests of positive and negative dengue infected patients show some distinguishable significant observations on clinical parameters by analyzing logistic regression, where it was found that arthralgia (*p* < 0.001) is the most prominent significant symptoms observed in ANA IIFA test positive dengue patients. To other significant clinical inspector such as myalgia (*p* = 0.023), body ache (*p* = 0.001) and skin rashes (*p* = 0.003) were also found to be prominently significant in the univariate analysis. But in case of multigrade logistic regression, it has been observed that arthralgia and body ache (*p* < 0.001) and myalgia (*p* = 0.006) were the most prominent significant parameters ANA IIFA positive dengue patients but skin rashes were unable to be significantly prominent (*p* = 0.423) within the comparison of ANA IIFA test positive and negative dengue infected patients (Table 2)

**Table 2.** Logistic regression analysis of Dengue-positive patients (in comparison to different risk factors.

	Regression analysis of ANA IIFA in dengue positive patients					
	Univariate logistic regression			Multivariate logistic regression		
Risk Factors	Odds Ratio	95% CI [lower-upper]	*P* value	Odds Ratio	95% CI [lower-upper]	*P* value
**Symptoms**						
Headache	1.59	0.64-3.91	0.309			
Nausea	2.03	0.86-4.80	0.104			
**Myalgia**	2.93	1.15-7.43	**0.023**	0.187	0.056-0.625	**0.006**
**Arthralgia**	22.90	6.49-80.71	**<0.001**	0.028	0.007-0.111	**<0.001**
Sore throat	2.12	0.85-5.30	0.105			
**Body ache**	4.37	1.83-10.41	**0.001**	0.109	0.036-0.324	**<0.001**
Abdominal pain	0.38	0.14-1.03	0.060			
Retro orbital pain	0.60	0.21-1.72	0.347			
Skin rash	2.99	1.45-6.16	**0.003**	0.680	0.265-1.745	0.423

As our previous observation on the ANA IIFA initial testing among the dengue positive infected patient strata represents some distinguishable clinical observations, so we have planned to analyze the comparison of ANA LIA confirmatory testing results. Some selected autoimmune specific antibodies among the dengue positive patients with the dengue negative patients to understand positive correlation of dengue infestation with respect to autoimmune diseases. In this observation study, we have selected seven autoimmune diseases such as SLE, Sjogren Syndrome, Crest, MCTD, PBC, Myositis, and Non-Rheumatic Diseases. It has been observed that MCTD (*p* = 0.005) and Myositis (*p* = 0.003) were found to be much more significant in dengue positive patients compared to dengue negative groups by univariate logistic regression. For this to autoimmune antibodies, we are also found to be highly significant during multivariate logistic analysis among both of these respective groups (*p* = 0.041 and 0.018 respectively). This observation of findings indicates that in dengue positive infestation these two autoimmune specific antibodies drastically increased and positively progress the autoimmune diseases (Table 3).

**Table 3.** Logistic regression analysis of ANA LIA tested dengue positive patients in comparison to different risk factors.

	Regression analysis of ANA LIA among dengue positive and negative groups					
	Univariate logistic regression			Multivariate logistic regression		
Antibodies of autoimmune disease	Odds Ratio	95% CI [lower-upper]	*P* value	Odds Ratio	95% CI [lower-upper]	P value
SLE	0.47	0.066-3.460	0.464			
Sjogren Syndrome	1.52	0.147-15.783	0.724			
Crest	1.43	0.114-18.076	0.779			
**MCTD**	14.01	2.197-89.215	**0.005**	0.112	0.014-0.911	**0.041**
PBC	0.30	0.036-2.573	0.275			
**Myositis**	18.37	2.746-122.944	**0.003**	0.082	0.010-0.657	**0.018**
Non-Rheumatic Disease	0.30	0.036-2.573	0.275			

## Discussion

When the health infrastructure is not well established, the dengue virus can have a serious negative effect on the health of individuals it infects and results in considerable expenses for the patient. Moreover, unusual presentations and a range of hazards are prevalent with the condition. Serological testing provides a confirmatory measure for dengue and should be interpreted cautiously, especially in cases of secondary dengue. However, clinical diagnosis remains the primary method of diagnosis. Although its usefulness in endemic areas is high, the dengue IgM antibody has limits that should be understood.

According to this research, DENV infection was associated with an increased incidence of autoimmune disorders overall. When examining specific autoimmune disorders, it was observed that dengue was linked to a higher incidence of MCTD and autoimmune myositis [13]. Our findings diverge significantly from those of Li et al., who claimed that dengue was linked to a variety of autoimmune conditions, such as multiple sclerosis, myasthenia gravis, autoimmune encephalomyelitis, SLE, post-infectious arthritis, systemic vasculitis, and primary adrenocortical insufficiency [13].

Furthermore, the clinical manifestation of dengue was strikingly comparable to signs of autoimmune disorders, including fever, skin rash, and thrombocytopenia. It is possible that some autoimmune disease patients in the Li et al. study were first misdiagnosed as having dengue during local epidemics and later acquired the right diagnosis [13].

There are various advantages to our study. First, there was a lengthy follow-up period with a sample size of 135 dengue positive patients and 126 suspected dengue controls. Secondly, the laboratory confirmed each case of dengue, reducing the potential for exposure status misclassification bias. Third, since the diagnosis of autoimmune myositis and MCTD was made using the gold standard technique for detecting ANAs, we thought it should be rather accurate.

In addition, LIA is more important for autoimmune diagnoses and has become easier and more reliable to process; therefore, all IIFA positive samples were retested under this technique using DFS70 as a systemic rheumatic illness exclusion marker. The presence of isolated DFS70 antibodies on LIA aids in the detection of false-positive IFA results. This guarantees increased dependability while excluding rheumatic illnesses. LIA is the best technique to determine whether DFS70 antibodies are present alone or in conjunction with other antibodies since it can detect a wide variety of antigens in a single test. But apart from autoimmune myositis and MCTD, no other autoimmune diseases were linked to an elevated risk of DENV infection, according to our research.

It has been demonstrated that the etiology of dengue involves autoimmunity [21]. In cases of severe dengue, autoantibodies generated during the virus may react with several self-antigens on endothelial cells, platelets, or coagulatory molecules. This reaction may lead to thrombocytopenia, coagulopathy, and vascular leakage [4,21,22]. Furthermore, the presence of autoantibodies in dengue-infected patients highlights a potential mechanism by which viral infections may trigger autoimmune responses. Molecular mimicry and bystander activation are plausible explanations for this phenomenon, where viral antigens mimic self-antigens, leading to an autoimmune response.

Autoantibodies produced by DENV experience titers that peak during the acute phase diminish during the convalescent period, and persist for multiple months [23]. Research results showed that within 180 days of a DENV infection, there was a substantial increase in the risk of autoimmune myositis and MCTD. Zika, Chikungunya, and West Nile are examples of mosquito-borne single-stranded RNA flaviviruses that have also been linked to autoimmune encephalomyelitis [24,25]. To elucidate the etiology, more research is necessary to determine the precise processes behind dengue-associated autoimmune encephalomyelitis. This study has certain limitation. Data on the serotypes of dengue infection and whether patients had primary or secondary infection were unavailable; therefore, further investigation on the association between autoimmune diseases and DENV serotype status could not be performed.

## Conclusion

When the illness progresses past the acute stage, dengue fever might present with uncommon signs and symptoms. Such uncommon presentations include myositis and MCTD, which can manifest as an autoimmune flare-up or as a de novo illness. In cases, such as these, it would appear prudent to expand the scope of studies to identify the disease in all dengue-infected patients, particularly in dengue endemic and epidemic areas, to prevent unintended delays in the diagnosis and treatment of autoimmune myositis and MCTD. This study showed that dengue was associated with an increased risk of autoimmune myositis and MCTD, a rare complication of dengue. The risk of other autoimmune diseases did not seem to increase after DENV infection. These findings suggest that clinicians should be aware of the potential for autoimmune complications in patients recovering from dengue. Further research is warranted to elucidate the mechanisms underlying this association and to develop strategies for early detection and management of autoimmune sequelae in dengue patients.

## Supplementary Material

Supplemental Material

Supplementary Table.docx

## Data Availability

The data that support the findings of this study are openly available in “figshare” at http://doi.org/10.6084/m9.figshare.25771896
