# Supplementary figures and images for: Detection and differentiation of antinuclear antibodies in serum of dengue suspected patients with or without systemic autoimmune disease in Kolkata, India
Source: Virulence. 2024 Sep 16;15(1):2400553. doi: 10.1080/21505594.2024.2400553 (PMC11407418; doi:10.1080/21505594.2024.2400553)

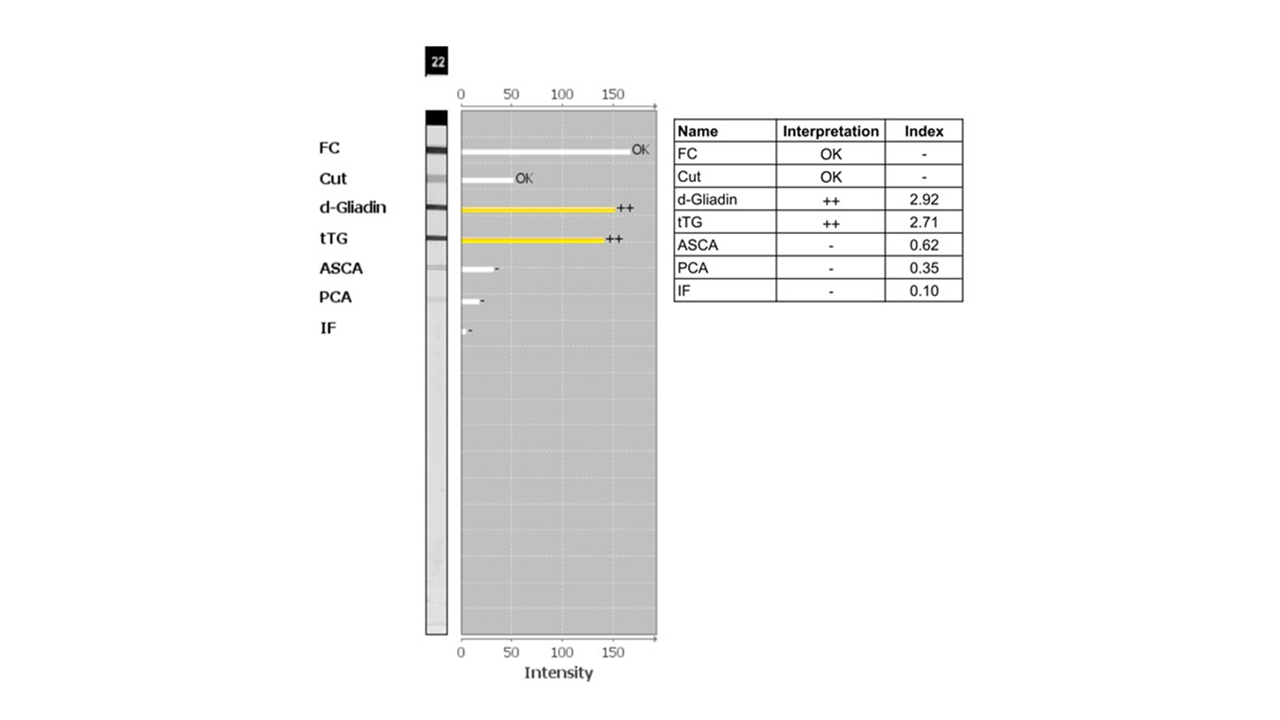

Supplement: Supplemental Material [file KVIR_A_2400553_SM0281.zip › Supplementary_Figure_S1300.tif]

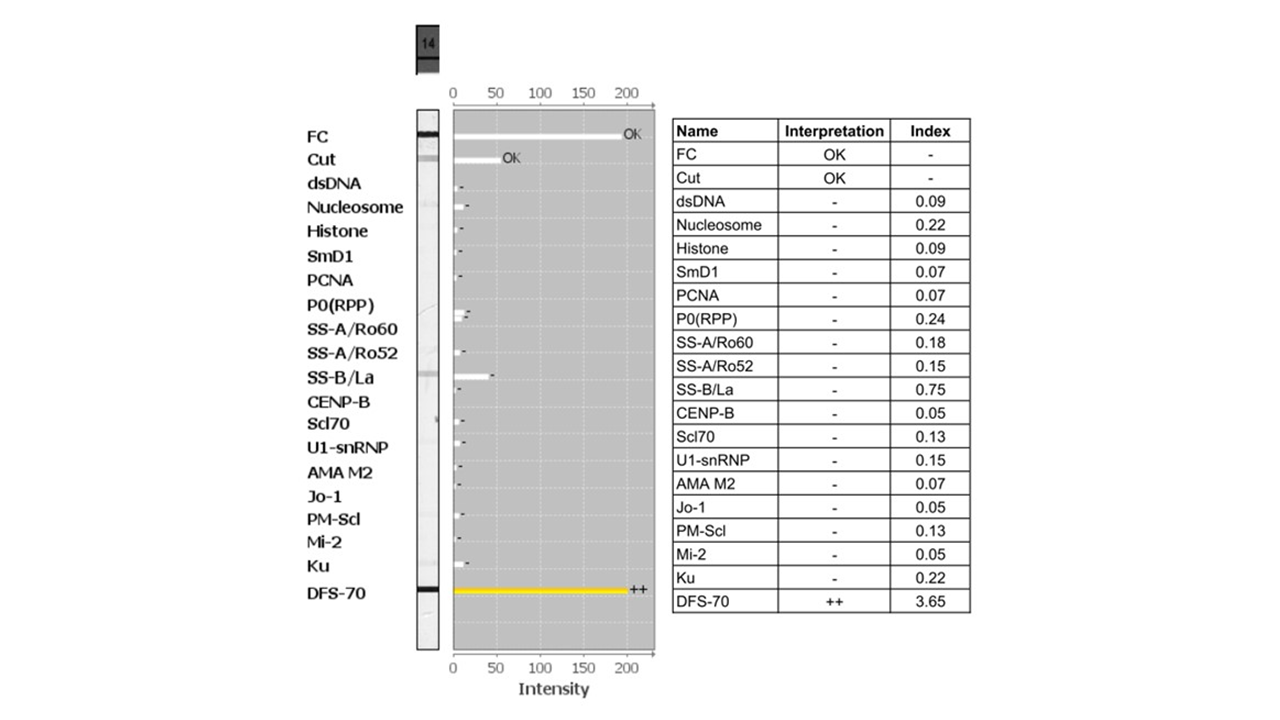

Supplement: Supplemental Material [file KVIR_A_2400553_SM0281.zip › Supplementary_Figure_S2300.tif]
