# Supplementary material for: Detection and differentiation of antinuclear antibodies in serum of dengue suspected patients with or without systemic autoimmune disease in Kolkata, India
Source: Virulence. 2024 Sep 16;15(1):2400553. doi: 10.1080/21505594.2024.2400553 (PMC11407418; doi:10.1080/21505594.2024.2400553)
Supplement: Supplementary Table.docx [file KVIR_A_2400553_SM5213.docx]

**Supplementary Table S1. Anti-nuclear antibodies associated with different autoimmune diseases**

| **Autoantibody Markers** | **Autoimmune Diseases** |
| --- | --- |
| dsDNA, Nucleosome, Histone, SmD1, PCNA, PO | SLE |
| SS-A/Ro 60 kDa, SS-A/Ro 52 kDa, SS-B/La | Sjögren's syndrome/SLE |
| CENP-B, Scl 70 | CREST/Scleroderma |
| U1-snRNP | MCTD |
| AMA M2 | PBC |
| Jo-1, PM-Scl, Mi-2, Ku | Myositis |
| DFS70 | Non-rheumatic autoimmune disease |
| PR3, MPO, GBM | Autoimmune vasculitis |
| d-Gliadin, tTG, ASCA, PCA, IF | Autoimmune gastritis |
| AMA-M2, Sp100, LKM1, gp210, LC1, SLA | Autoimmune hepatitis |

Abbreviation: SmD1, smith D1 antibody; PCNA, Proliferating cell nuclear antigen; PO, anti-ribosomal P antibody; SS-A/Ro, anti–Sjögren's-syndrome-related antigen A autoantibodies; SS-B/La, anti-Sjögren's syndrome type B antibodies; CENP-B, anti-centromere protein B antibodies; Scl 70, scleroderma 70; snRNP, smaller nuclear ribonucleoprotein; AMA, Anti-mitochondrial antibodies; Jo-1, anti-histidyl-tRNA synthetase; PM-Scl, polymyositis and scleroderma; DFS70, anti-dense fine speckled 70; PR3, Proteinase 3; MPO, myeloperoxidase; GBM, anti-glomerular basement membrane; tTg, Tissue transglutaminase; ASCA, Anti-Saccharomyces cerevisiae antibodies; PCA, Purkinje cell autoantibodies; IF, interferons; LKM1, Liver kidney microsome type 1; gp210, glycoprotein 210; LC1, liver cytosolic antigen type 1; SLA, soluble liver antigen; CREST, calcinosis, Raynaud phenomenon, esophageal dysmotility, sclerodactyly, and telangiectasia; PBC, primary biliary cirrhosis
